# Supplementary material for: Ovarian Morphology in Non-Hirsute, Normo-Androgenic, Eumenorrheic Premenopausal Women from a Multi-Ethnic Unselected Siberian Population
Source: Diagnostics (Basel). 2024 Mar 22;14(7):673. doi: 10.3390/diagnostics14070673 (PMC11012196; doi:10.3390/diagnostics14070673)
Supplement: Supplementary file 1 [file diagnostics-14-00673-s001.zip › diagnostics-2892322-supplementary.pdf]

**Table S1.** Characteristics of healthy women included to the study population.

| Parameter | Total<br>n= 408 | Caucasians<br>n=285 | Asians<br>n=123 | p-value |
|---|---|---|---|---|
| | | Mean ± SD<br>Median (Lower Q; Upper Q) | | |
| Age, years | 34.32±5.96<br>35.00 (30.00;39.00) | 34.04±6.01<br>34.00 (30.00;39.00) | 34.98±5.81<br>35.00 (31.00;40.00) | $p_U$=0.680 |
| | | Anthropometry and vital signs<br>Mean ± SD<br>Median (Lower Q; Upper Q) | | |
| WC, cm | 74.14±8.85<br>73.00 (67.00;80.00) | 73.86±8.95<br>73.00 (67.00;80.00) | 74.80±8.63<br>74.00 (68.00;81.00) | $p_U$=0.649 |
| BMI, kg/m$^2$ | 23.79±3.30<br>23.70 (21.15;26.60) | 23.90±3.29<br>23.90 (21.20;26.70) | 23.54±3.31<br>23.70 (20.90;26.30) | $p_U$=0.916 |
| Systolic blood pressure, mm Hg | 119.51±11.52<br>119.00 (112.00;126.00) | 119.40±11.02<br>120.00 (112.00;125.00) | 119.75±12.64<br>117.00 (111.00;126.00) | $p_U$=0.066 |
| Diastolic blood pressure, mm Hg | 76.51±8.61<br>76.00 (70.00;81.500) | 76.00±8.39<br>76.00 (70.00;81.00) | 77.71±9.05<br>77.00 (71.00;83.00) | $p_U$=0.307 |
| | | Education n/N (%) | | ***$p_{KW}$<0.001*** |
| Doctoral degree | 27/408 (6.62%) | 15/285 (5.26%) | 12/123 (9.76%) | $p_z$=0.093 |
| Master's degree | 286/408 (70.10%) | 185/285 (64.91%) | 101/123 (82.11 %) | ***$p_z$=0.001*** |
| Incomplete high school | 16/408 (3.92%) | 13/285 (4.56%) | 3/123 (2.44%) | $p_z$=0.311 |
| Bachelor's degree | 58/408 (14.22%) | 52/285 (18.25%) | 6/123 (4.88%) | ***$p_z$<0.001*** |
| Some college | 1/408 (0.25%) | 1/285 (0.35%) | 0/123 (0.00%) | $p_z$=0.511 |
| High school or equivalent | 14/408 (3.43%) | 13/285 (4.56%) | 1/123 (0.81%) | $p_z$=0.056 |
| Middle school only | 5/408 (1.23%) | 5/285 (1.75%) | 0/123 (0.00%) | $p_z$=0.140 |
| Elementary school | 0/408 (0.00%) | 0/285 (0.00%) | 0/123 (0.00%) | NA |
| No degree | 1/408 (0.25%) | 1/285 (0.35%) | 0/123 (0.00%) | $p_z$=0.511 |
| | | Occupation, n/N (%) | | ***$p_{KW}$<0.001*** |
| Legislators. senior officials and managers | 9/408 (2.22%) | 8/285 (2.83%) | 1/123 (0.82%) | $p_z$=0.511 |
| Professionals | 179/408 (44.20%) | 108/285 (38.16%) | 71/123 (58.20%) | $p_z$=0.206 |
| Technicians and associate professionals | 78/408 (19.26%) | 62/285 (21.91%) | 16/123 (13.11%) | ***$p_z$<0.001*** |
| Office clerks | 55/408 (13.58%) | 33/285 (11.66%) | 22/123 (18.03%) | ***$p_z$=0.039*** |
| Service workers and shop and market sales | 21/408 (5.19%) | 13/285 (4.59%) | 8/123 (6.56%) | $p_z$=0.084 |
| Skilled agricultural and fishery workers | 1/408 (0.25%) | 1/285 (0.35%) | 0/123 (0.00%) | $p_z$=0.511 |
| Craft and related trades workers | 41/408 (10.12%) | 38/285 (13.43%) | 3/123 (2.46%) | ***$p_z$=0.001*** |
| Plant and machine operators and assemblers | 8/408 (1.98%) | 8/285 (2.83%) | 0/123 (0.00%) | $p_z$=0.059 |
| Elementary occupations | 13/408 (3.21%) | 12/285 (4.24%) | 1/123 (0.82%) | $p_z$=0.072 |
| | | Marital status n/N (%) | | $p_{KW}$=0.890 |
| Single | 97/408 (23.89%) | 68/285 (23.86%) | 29/123 (23.97%) | |
| Married | 220/408 (54.19%) | 152/285 (53.33%) | 68/123 (56.20%) | |
| Living with another | 38/408 (9.36%) | 30/285 (10.53%) | 8/123 (6.61%) | |
| Separated | 5/408 (1.23%) | 4/285 (1.40%) | 1/123 (0.83%) | |
| Divorced | 35/408 (8.62%) | 23/285 (8.07%) | 12/123 (9.92%) | |
| Widowed | 8/408 (1.97%) | 6/285 (2.11%) | 2/123 (1.65%) | |
| Would rather not say | 3/408 (0.74%) | 2/285 (0.70%) | 1/123 (0.83%) | |

| | | | | |
|---|---|---|---|---|
| | Menstrual and reproductive history<br>Mean ± SD<br>Median (Lower Q; Upper Q) | | | |
| Age at menarche, years | 13.28±1.35<br>13.00 (12.00;14.00) | 13.22±1.31<br>13.00 (12.00;14.0) | 13.41±1.43<br>13.00 (12.00;14.00) | $p_U$=0.223 |
| Average menstrual cycle length, days | 27.71±2.11<br>28.00 (27.99;29.00) | 27.61±2.16<br>28.00 (27.00;28.00) | 27.93±1.97<br>28.00 (27.00;30.00) | $p_U$=0.251 |
| Parity of pregnancies | 2.36±2.15<br>2.00 (1.00;3.00) | 2.37±2.19<br>2.00 (1.00;4.00) | 2.33±2.04<br>2.00 (1.00;3.00) | $p_U$=0.375 |
| mFG score | 0.54±0.96<br>0.00 (0.00;1.00) | 0.62±1.02<br>0.00 (0.00;1.00) | 0.37±0.78<br>0.00 (0.00;0.00) | ***$p_U$=0.001*** |
| | | Hormones<br>Mean ± SD<br>Median (Lower Q; Upper Q) | | |
| Prolactin, mIU/ml | 333.91±153.57<br>307.00 (218.00;438.50) | 303.38±134.08<br>277.00 (202.00;382.00) | 404.64±172.07<br>399.00<br>(269.00;509.00) | ***$p_U$=0.001*** |
| TSH, mIU/ml | 1.56±0.73<br>1.50 (1.00;2.00) | 1.52±0.73<br>1.40 (1.00;1.90) | 1.66±0.72<br>1.60 (1.20;2.10) | $p_U$=0.996 |
| LH, mIU/ml | 7.23±10.02<br>5.30 (3.20;7.50) | 7.52±10.16<br>5.60 (3.30;7.90) | 6.57±9.67<br>4.80 (3.20;7.00) | $p_U$=0.533 |
| FSH. mIU/ml | 5.86±3.22<br>5.40 (3.8;7.0) | 5.96±3.22<br>5.50 (4.00;7.00) | 5.67±3.22<br>5.10 (3.60;6.90) | $p_U$=1.00 |
| TT, ng/dl | 25.02±13.74<br>24.69<br>(14.93;33.61) | 26.22±14.68<br>25.70<br>(16.05;34.83) | 21.78±10.65<br>22.09<br>(13.64;29.99) | ***$p_U$<0.001*** |
| SHBG, nmol/l | 86.23±56.21<br>70.35 (47.00;105.70) | 90.48±59.98<br>71.00 (51.40;115.00) | 76.38±45.00<br>65.60 (43.50;96.80) | ***$p_U$<0.001*** |
| FAI | 1.35±1.05<br>1.15 (0.59;1.83) | 1.42±1.17<br>1.19 (0.59;1.88) | 1.19±0.70<br>1.07 (0.57;1.62) | ***$p_U$<0.001*** |
| DHEAS, μg/dl | 160.52±65.36<br>155.00 (113.00;202.00) | 164.65±68.89<br>158.00 (118.00;210.00) | 150.87±55.32<br>145.00<br>(106.00;187.00) | ***$p_U$=0.006*** |
| 17OHP, nmol/l | 5.50±3.37<br>5.15 (2.50;7.00) | 5.63±3.35<br>5.40 (2.80;7.00) | 5.21±3.41<br>5.00 (2.10;7.00) | $p_U$=0.810 |
| AMH, ng/ml | 2.85± 2.21<br>2.20 (1.10;4.40) | 2.91±2.20<br>2.30 (1.10;4.50) | 2.71±2.22<br>1.90 (0.57;1.62) | $p_U$=0.900 |

Abbreviations: WC is waist circumference, BMI is body mass index, mFG score is modified Ferriman-Gallwey score, NA- not applicable; U, Z, KW – U-test; Z test; Kruskal-Wallis ANOVA test, respectively
